# Autoimmune Thyroid Disorders

**DOI:** 10.1155/2013/509764

**Published:** 2013-06-26

**Authors:** M. A. Iddah, B. N. Macharia

**Affiliations:** ^1^Department of Biomedical Science and Technology, Maseno University, P.O. Box 333-40105, Maseno, Kenya; ^2^Department of Medical Laboratory, Moi Teaching and Referral Hospital, P.O. Box 3-30100, Eldoret, Kenya; ^3^Department of Human Pathology, School of Medicine, Moi University, P.O. Box 4606-30100, Eldoret, Kenya

## Abstract

*Purpose of Review*. Studies have been published in the field of autoimmune thyroid diseases since January 2005. The review is organized into areas of etiology, autoimmune features, autoantibodies, mechanism of thyroid cell injury, B-cell responses, and T-cell responses. Also it reviews the diagnosis and the relationship between autoimmune thyroid disease, neoplasm, and kidney disorders. *Recent Findings*. Autoimmune thyroid diseases have been reported in people living in different parts of the world including North America, Europe, Baalkans, Asia, Middle East, South America, and Africa though the reported figures do not fully reflect the number of people infected per year. Cases are unrecognized due to inaccurate diagnosis and hence are treated as other diseases. However, the most recent studies have shown that the human autoimmune thyroid diseases (AITDs) affect up to 5% of the general population and are seen mostly in women between 30 and 50 years. *Summary*. Autoimmune thyroid disease is the result of a complex interaction between genetic and environmental factors. Overall, this review has expanded our understanding of the mechanism involved in pathogenesis of AITD and the relationship between autoimmune thyroid disease, neoplasm, and kidney disease. It has opened new lines of investigations that will ultimately result in a better clinical practice.

## 1. Introduction

The principal diseases of the human thyroid gland are goiter (diffuse or nodular), hyperthyroidism, hypothyroidism, autoimmune thyroiditis, and neoplasm [1]. The thyroiditis types cause inflammation of thyroid tissue and can release preformed hormone from the colloid space, causing thyrotoxicosis, which is transient and followed by recovery or development of hypothyroidism. In acute and subacute thyroiditis, thyroid tenderness and neck pain are often present. On the other hand, silent thyroiditis is devoid of the local symptoms [2]. 

In the USA and Canada, the extrapolated prevalences are 5,873,108 and 650,157, respectively. In Austria and Belgium, the prevalences are 163,495 and 206,965, respectively. In Bosnia and Macedonia, the prevalences are 8,152 and 40,801, respectively. For China and India, the prevalences are 25,976,952 and 21,301,412, respectively, while in Egypt and Iran they are 1,522,348 and 1,350,064, respectively. South Africa has a prevalence of 888,969 [3]. The annual incidence of Hashimoto's thyroiditis worldwide is estimated to be 0.3–1.5 cases per 1000 persons, whereas Graves' disease is estimated at about 5 per 10,000 people [4]. 

The human AITDs broadly include Graves' disease (GD) and Hashimoto's thyroiditis (HT) which are the most common causes of thyroid gland dysfunctions and nonendemic goiter [4]. These conditions arise due to complex interactions between environmental and genetic factors [5] and are characterized by reactivity to self-thyroid antigens which are expressed as distinctive inflammatory or antireceptor autoimmune diseases [6, 7]. Among the major AITD susceptibility genes that have been identified and characterized is the HLA-DR gene locus, as well as non-MHC genes including the CTLA-4, CD40, PTPN22, thyroglobulin, and TSH receptor genes [8]. The major environmental triggers of AITD include iodine, medications, infection, smoking, stress, and genetic predisposition to AITD which lead to novel putative mechanisms by which the genetic-environmental interactions may lead to the development of thyroid autoimmunity [9]. 

The first pathological features of autoimmune thyroiditis were described in 1912 [10] when patients with goiter exhibited diffuse lymphocyte infiltration, atrophy of follicular cells, presence of granulated thyrocytes (oncocytic cells or Hurtle's cells), and fibrosis in the histological pictures of their thyroid tissues [10]. The Hashimoto's thyroiditis disorder is directed against thyroid antigens and is the most common cause of hypothyroidism [11]. The incidence is 0.3 to 1.5 per 1000 persons per year, and it is 4 to 10 times more common in women than in men [8, 11, 12]. Hashimoto's thyroiditis is more prevalent in areas with a high dietary iodinized salt intake, and smoking increases the risk [8]. Goiter can be seen on presentation, but thyroid atrophy is more common. Hashimoto's thyroiditis is associated with other endocrine diseases in polyglandular autoimmune failure syndrome (Addison's disease, type 1 *diabetes mellitus*, and hypogonadism) [8]. The diagnosis is made by clinical features, elevated TSH, low thyroid hormone, and the presence of antithyroid peroxidase antibodies (anti-TPO) [13]. 

Graves' disease, on the other hand, involves the binding of autoantibodies to TSH receptor which leads to stimulation. It is the most common cause of thyrotoxicosis [14]. Receptor activation stimulates thyrocyte growth and function [15]. The disease is more common in Whites and Asians, and the incidence is lower in African Americans, and female-to-male ratio is 3.5 : 1 [16]. It is more common in patients with a family history of thyroid disease, especially Graves' disease. Graves' disease features include swelling over the anterior shin (pretibial myxedema), thyroid eye disease (prominence of eyes, lid lag, globe lag, exophthalmos, lid edema, chemosis, and extraocular muscle weakness), and increased pigmentation and vitiligo. Thyroid ophthalmopathy is present in about 50% of Graves' patients. Smoking is a risk factor, and therapeutic options include local measures to combat inflammation—glucocorticoids, plasmapheresis, and immune suppressants as well as orbital radiation, decompressive surgery, and thyroid ablation [2]. 

## 2. Etiology

The etiology of AITD is multifactorial. Susceptibility to the disease is determined by a combination of immune mechanism, genetics, and environmental (iodine, infection, and stress) and constitutional factors.

## 3. Immune Mechanisms

A variety of immune mechanisms may be involved in the pathogenesis of Graves' hyperthyroidism. The major mechanisms for which there is some evidence are molecular mimicry (specificity crossover), thyroid-cell expression of HLA (human leukocyte-associated) molecules (antigens), and bystander activation [17].

## 4. Molecular Mimicry

Molecular mimicry implies structural similarity between some infectious or other exogenous agent and human proteins, such that antibodies and T cells activated in response to the exogenous agent react with the human protein, in this instance one or more thyroid proteins. As an example, in an analysis of 600 monoclonal antibodies raised against a large variety of viruses, 4 percent of the monoclonal antibodies cross-reacted with uninfected tissues [18].

## 5. Thyroid Cell Abnormal Expression of HLA II Molecules

Thyroid epithelial cells from patients with autoimmune thyroid disease (including Graves' disease) but not normal subjects express MHC class II molecules, notably HLA-DR molecules [19]. This expression could be the direct result of viral or other infections of thyroid epithelial cells, or it may be induced by cytokines such as interferon-gamma produced by T cells that have been attracted to the gland either by an infection or directly because of the presence of thyroid antigens [20].

Class II molecule expression provides a mechanism for presentation of thyroid antigens to and activation of autoreactive T cells, with the potential for persistence of thyroid disease [20]. Several experimental observations provide support for this hypothesis: induction of class II molecules on thyroid epithelial cells by interferon-gamma can induce autoimmune thyroiditis in susceptible mice [21]; viruses can directly induce class II molecule expression on thyroid cells, independent of cytokine secretion [20, 22]; thyroid epithelial cells expressing class II molecules can present viral peptide antigens to cloned T-cells [23]; thyroid antigen-specific T cell clones in normal rats react specifically with cloned autologous thyroid cells in the absence of more conventional antigen-presenting cells [24]; and an animal model of Graves' disease induced by cells expressing the TSHR is only effective when the cells also express MHC class II antigens [25, 26]. These findings strongly support the view that an insult, such as infection, may induce class II molecule expression on human thyroid cells and that these cells then may act as antigen-presenting cells to initiate an autoimmune response [20]. 

The expression of a T-cell costimulator molecule, CD40, on thyroid epithelial cells indicates that costimulatory molecules are available for this action. In addition, intrathyroidal dendritic cells and B cells may also serve as potent antigen-presenting cells [27, 28]. The description of hyperthyroidism in mice immunized with fibroblasts coexpressing class II molecules and human TSH receptors provides further evidence that cells need not be “professional” antigen-presenting cells to present antigen so long as they can acquire the ability to express class II molecules [29].

## 6. Bystander Activation

In order for HLA class II antigen expression and presentation of antigens to be realized, there must be a local insult to initiate the responses. As mentioned above, this may take the form of a direct insult to the thyroid by a viral infection of the thyroid cells or of immune cells. Even the arrival of activated T cells within the thyroid gland may perhaps initiate such a series of events in a susceptible subject with the appropriate immune repertoire [30]. Evidence shows that such bystander activation of local T cells, which may not be thyroid specific, may exert via cytokines a marked activation effect on resident thyroid-specific T cells. Evidence for such bystander effects has been obtained in an animal model of viral-induced autoimmune insulitis and in experimental autoimmune thyroiditis [31]. 

## 7. Precipitating and Predisposing Factors for Graves' Disease

Several factors that predispose to or initiate Graves' hyperthyroidism have been proposed and include genetic susceptibility, infection, stress, sex steroids, smoking, pregnancy, and drugs as reviewed in the sections that follows [32].

## 8. Genetic Susceptibility

There is abundant epidemiologic evidence for genetic susceptibility to Graves' hyperthyroidism and chronic autoimmune thyroiditis [14, 33]. The diseases cluster in families and are more common in women. The concordance rate in monozygotic twins is 20 to 40 percent [34]. The sibling recurrence rate for Graves' disease exceeds 10.0 [35]. There is an association between autoimmune thyroid disease and certain alleles of CTLA-4 (cytotoxic T lymphocyte-antigen/associated protein 4). As an example, in one study of 379 patients with Graves' hyperthyroidism in the United Kingdom, 42 percent had a particular allele (G allele) of the CTLA-4 gene, as compared with 32 percent of 363 normal subjects [36]. There is an association with certain alleles of HLA on chromosome 6. As an example, a study of Caucasian patients in North America found that HLA-DRB1*08 and DRB3*0202 were associated with the disease and that DRB1*07 was protective [5, 37, 38]. 

## 9. Infection

If infection was the cause of Graves' hyperthyroidism, an identifiable agent should be present in the majority of patients and it should be possible to induce the disease by transferring the agent. Possible infections of the thyroid gland itself (subacute thyroiditis and congenital rubella) have been associated with thyroid autoimmune disease and could initiate class II molecule expression [39]. Hepatitis C infection is a well-recognized precipitator of autoimmune thyroid disease when treated with interferon therapy. There is, however, no evidence that these or any other infections or exposures lead directly to autoimmune thyroid disease [39–41]. 

## 10. Stress

As compared with normal subjects or patients with toxic nodular goiter, patients with Graves' hyperthyroidism more often give a history of some type of psychologic stress in particular negative life events such as loss of a spouse before the onset of their hyperthyroidism [42–44]. In general, stress appears to induce a state of immune suppression, possibly mediated by the actions of cortisol on immune cells. Suppression of stress may be followed by rebound immunologic hyperactivity. Such a response could precipitate autoimmune thyroid disease in genetically susceptible subjects [42–44].

## 11. Sex Steroids

More women develop Graves' hyperthyroidism than men, with a ratio of approximately 7 : 1; an effect that is often said to be mediated in some way by more estrogen or less testosterone [45]. There is a large body of evidence that moderate amounts of estrogen enhance immunologic reactivity to self-antigens [46, 47]. However, it is just as likely that the X chromosome is the source of the enhanced susceptibility rather than sex steroids since the susceptibility continues after the menopause. For example, X-chromosome inactivation has been associated with autoimmune thyroid disease [45].

## 12. Smoking

Smoking is a risk factor for Graves' hyperthyroidism (relative risk approximately 2.0) and an even stronger risk factor for Graves' ophthalmopathy [48–50].

## 13. Pregnancy

Graves' disease is uncommon during pregnancy because hyperthyroidism is associated with reduced fertility and increased pregnancy loss. In addition, pregnancy is a time of immune suppression so that the disease tends to improve as pregnancy progresses. During pregnancy, both T-cell and B-cell functions are diminished, and the rebound from this immunosuppression may contribute to the development of postpartum thyroid disease [51]. It has also been suggested that fetal microchimerism (the presence of fetal cells in maternal tissue) might play a role in the development of postpartum autoimmune thyroid disease [52]. Up to 30 percent of young women give a history of pregnancy in the 12 months before the onset of Graves' disease, indicating that postpartum Graves' disease is a surprisingly common presentation and that pregnancy is a major risk factor in susceptible women [53].

## 14. Drugs

Iodine and iodine-containing drugs such as amiodarone may precipitate Graves' disease, or a recurrence of Graves' disease, in a susceptible individual [48, 54]. Iodine is most likely to precipitate thyrotoxicosis in an iodine deficient population simply by allowing the TSHR-Ab to be effective in stimulating the production of thyroid hormone. Whether there is any other precipitating event is unclear. Iodine and amiodarone may also damage thyroid cells directly and release thyroid antigens to the immune system [55].

## 15. Predisposing and Precipitating Factors for Hashimoto's Thyroiditis

 Infection, stress, sex steroids, pregnancy, iodine intake, and radiation exposure are the known possible precipitating factors for Hashimoto's thyroiditis [56]. Fetal microchimerism within the maternal thyroid is also a possibility [52, 56–59].

## 16. Genetic Susceptibility

There is genetic susceptibility to Hashimoto's thyroiditis, and much has been learned in recent years concerning the susceptibility genes for this disorder in particular and for autoimmune thyroid disease in general [60]. Evidence for genetic susceptibility to Hashimoto's thyroiditis includes the following observations.

The disease clusters in families, sometimes alone and sometimes in combination with Graves' disease [61]. The sibling recurrence risk is >20 [35]. The concordance rate in monozygotic twins is 30 to 60 percent despite random combinations of T-cell receptor and antibody V genes at the time of recombination [62]. There is an association, albeit relatively weak, with certain HLA alleles such as DR3. There is linkage to certain alleles of the gene for CTLA-4. The thyroglobulin gene has been linked to autoimmune thyroid disease and has been suggested to code for Tg forms with different immune reactivity [15].

## 17. Infection

No infection is known to cause or even be closely associated with Hashimoto's thyroiditis in humans [63], although thyroiditis can be induced in experimental animals by certain viral infections [57]. Patients with subacute granulomatous thyroiditis (presumed to be a viral infection) and congenital rubella may have thyroid antibodies for a few months after their illnesses, and the infections could initiate expression of MHC class II molecules in the thyroid gland. However, neither disorder is known to be commonly followed by chronic thyroiditis although evidence of thyroid autoimmunity may persist [64].

## 18. Stress

Stress of various types has been linked to Hashimoto's thyroiditis. The proposed mechanisms include induction of immune suppression by nonantigen-specific mechanisms, perhaps due to the effects of cortisol or corticotropin-releasing hormone on immune cells, followed by immune hyperactivity leading to autoimmune thyroid disease [58]. 

## 19. Sex Steroids and Pregnancy

More women than men have Hashimoto's thyroiditis, suggesting a role for sex steroids. However, older women may be more likely to have Hashimoto's thyroiditis than younger women, suggesting that the presence or absence of estrogen may not be the important factor [65].

Another possible explanation for female predominance is skewed X-chromosome inactivation, which was found in 34 percent of female twins with autoimmune thyroid disease and only 11 percent of controls [45, 66]. It is possible that the self-antigens on the inactivated X-chromosome might not be expressed sufficiently to allow tolerance. During pregnancy, there is a marked increase in CD4+ CD25+ regulatory T cells which lead to diminished functions of both T cells and B cells, and the rebound from this immunosuppression is thought to contribute to the development of postpartum thyroiditis [65]. Pregnancy-associated immune suppression is associated with a shift to Th_2_ T cells and a shift in cytokine profiles [65].

A variety of local factors at the immune cell-trophoblast interface are also known to be important modulators of immune function in pregnancy. The trophoblast cells located in the placenta, and subject to maternal immune surveillance serve as physical barriers between mother and fetus and have been shown to express several immune modulating molecules, such as HLA-G, FasL, and indoleamine 2,3-dioxygenase as well as secreting a variety of cytokines [67]. HLA-G is one of the members of the MHC class I family and is known to inhibit natural killer cell function and dendritic cell maturation. Fas ligand interacts with Fas antigen and induces apoptotic cell death of fetal antigen-reactive maternal lymphocytes. Indoleamine 2,3-dioxygenase, which catalyzes tryptophane in lymphocytes, has proven to be critical in the maintenance of allogeneic pregnancy in mouse [68]. Other than these local modulators, progesterone produced by the placenta affects cytokine profiles across the whole maternal immune system. Approximately 20 percent of patients with postpartum thyroiditis go on to develop classical Hashimoto's disease in later years [69].

## 20. Iodine Intake

Mild iodine deficiency is associated with lower prevalences of Hashimoto's disease and hypothyroidism, while excessive intake is associated with a higher prevalence [70]. As an example, in China, autoimmune thyroiditis was found in 0.3 percent of those with mildly deficient iodine intake and 1.3 percent of those with excessive iodine intake [56].

## 21. Radiation Exposure

 Following the tragic Chernobyl nuclear accident, the exposed children developed a high frequency of thyroid autoantibodies [71]. All the evidence suggests that the presence of thyroid antibodies increases the risk of developing thyroid dysfunction [4, 72]. Whether background radiation to which we are all exposed has any role in susceptibility to autoimmune thyroid disease is unknown. In a population-based study of 4299 subjects, 160 had an occupational exposure to ionizing radiation, nearly 60 percent of the subjects worked in a nuclear power plant, while the rest were either medical or laboratory workers. Ten percent of the female subjects with radiation exposure met criteria for autoimmune thyroid disease (anti-TPO antibodies greater than 200 IU/mL and hypoechogenicity on ultrasound) compared to 3.4 percent of those without an exposure. Subjects with greater than five years of exposure to ionizing radiation were particularly at high risk [73]. 

## 22. Fetal Microchimerism

Fetal cells have been identified within maternal thyroid glands in patients with autoimmune thyroid disease. Such cells may initiate graft versus host reactions with the thyroid gland and play a significant role in the development of Hashimoto's thyroiditis. To date, however, this remains hypothetical.

## 23. Autoimmune Features

All forms of thyroid autoimmunity are associated with a lymphocytic infiltrate in the thyroid. These lymphocytes are largely responsible for generating both T- and B-cell-mediated autoreactivity. Other sites such as thyroid draining lymph nodes and bone marrow may also contain thyroid autoreactive lymphocytes in AITD. The initial autoimmune response by CD4+ T cells appears to upregulate the secretion of interferon-gamma resulting in enhancing the expression of MHC II molecules on thyrocytes. This most likely triggers expansion of autoreactive T cells and gives rise to the characteristic inflammatory response, and as the disease progresses, thyrocytes are targeted for apoptosis resulting in hypothyroidism. Another contributing factor to the observed hypothyroidism in Hashimoto's thyroiditis patients could be the circulating TSH inhibitory antibodies. Graves' disease on the other hand represents the other end of spectrum wherein the patients suffer from hyperthyroidism. The activation of thyroid specific CD4+ T cells leads to the recruitment of autoreactive B cells and the mounting of thyroid stimulatory immune response via antithyroid antibodies [74]. 

## 24. Autoantibodies

### 24.1. Thyroid Peroxidase (TPO) Antibodies

Thyroid peroxidase (TPO) antibodies are the key thyroid enzyme catalyzing both the iodination and coupling reaction for the synthesis of thyroid hormone. It is membrane bound and found in the cytoplasm and in high concentration on the apical microvillar surface of thyrocytes. It is of mol wt between 100 to 105-kDa and previously was known as thyroid microsomal antigen [75]. Multiple T- and B-cell epitopes exist within the molecule, and the antibody response to TPO is restricted at the level of the germ line heavy and light chain variable (V) region [76].

Anti-TPO autoantibodies are found in over 90% of patients with autoimmune hypothyroidism and Graves' disease. Together with thyroglobulin (TG) antibodies, these are the predominant antibodies in autoimmune hypothyroidism (AH). Anti-TPO antibodies are mainly of the IgG class 1 and IgG4 subclasses in excess [77–79]. 

### 24.2. Thyroglobulin (TG) Antibodies

Thyroglobin (TG) is a 660-kDa glycoprotein composed of two identical subunits of 330 kDa each. It is secreted by the thyroid follicular cells into the follicular lumen and stored as a colloid substance within the thyroid follicles. Each TG molecule has around 100 tyrosine residues, a quarter of which are iodinated. These residues couple to for triiodothyronine (T3) and thyroxine (T4). The sequence of human TG has been determined [80]. When TSH stimulates the thyroid cell, TG is endocytosed and hydrolyzed in lysosome releasing T3 and T4. The exact location of T- and B-cell epitopes within TG is uncertain [81].

Thyroglobulin autoantibodies are found in less than 60% of patients with lymphocytic thyroiditis and 30% of Graves' disease patients. They are polyclonal and mainly of IgG class with all four subclasses represented. TSH regulates the cell surface expressions of TPO and TG altering the transcription of these two proteins, possibly at the gene promoter level. These effects are mimicked by autoantibodies (both blocking and stimulating) in sera of patients with Graves' disease [82].

### 24.3. Thyroid Stimulating Hormone Receptor (TSH-R) Antibodies

Thyroid stimulating hormone receptor (TSH-R) is the prime autoantigen in Graves' disease and atrophic thyroiditis. It is located on the basal surface of thyroid follicular cells [83]. In Graves' disease, thyroid stimulating antibodies (TSAbs) bind to the receptor and stimulate the thyroid cell to produce excessive amount of thyroid hormones resulting in hyperthyroidism. In patients with atrophic thyroiditis, the major antibody is the TSH to its receptor, thus preventing stimulation of thyroid cell. This results in diminished thyroid hormone output, atrophy of thyroid gland, and the clinical state of hypothyroidism [83, 84].

### 24.4. Mechanism of Thyroid Cell Injury

Several antibody and cell-mediated mechanisms contribute to thyroid injury in autoimmune thyroid disease. In general, in cases of Hashimoto's thyroiditis, the expressions of death receptor CD95 and death receptor ligands CD95L in the thyroid tissue appear to be much higher compared to their normal counterparts. Also the expression of positive effectors of apoptosis and caspases 3 and 8 as well as Bax and Bak appear to be relatively high in thyroiditis samples as compared to controls. This expression pattern supports enhanced apoptosis as the mechanism underlying the loss of thyrocytes in Hashimoto's thyroiditis. In Graves' disease, there is highly elevated expression of negative modulators of apoptosis (cFLIP, Bcl-2, and Bcl-XL). This supports the role for apoptosis inhibitory mechanism. Although in both cases there is significant expression of Fas/CD95 and its ligand, only in Hashimoto's thyroiditis, the thyrocytes undergo apoptosis. The role of cytokines in the development of autoimmune disorders has also been explained [85]. In case of Hashimoto's thyroiditis, a TH1 disease, the cytokine interferon-gamma appears to play a crucial role in the pathology of the disease by enhancing the expression of caspases and thereby sensitizing cells to FAS-mediated apoptosis. In contrast, in the TH2-mediated Graves' disease, the cytokines IL4 and IL-10 regulate the expressions of two anti-apoptotic proteins Bcl-XL and cFLIP, which offers resistance to Fas-mediated apoptosis. This proves the necessary modulatory roles played by the TH1 and TH2 cytokines in the development of autoimmune disorders [74].

### 24.5. B Cell Responses

Thyroglobin (TG) and TPO antibodies occur in very high concentration in patients with Hashimoto's thyroiditis and primary myxedema. These antibodies are less common but still frequent in Graves' disease, whereas TPO rather than TG antibodies are frequent in postpartum thyroiditis [86]. Both of the antibodies show partial restriction to the IgG4 subclass [76]. TG antibodies usually mediate antibody-mediated cytotoxicity (ADCC), whereas TPO antibodies form terminal complement complexes within the thyroid gland. Cell-mediated injury may be necessary for TPO antibodies to gain access to their antigen and become pathogenic [87].

### 24.6. T-Cell Responses

Both CD4+ and CD8+ T cells occur in thyroid lymphocytic infiltrate with a preponderance of CD4+ cells. There is an increase in activated T-cell expressing markers like HLA-DR. Cytokines including IL-2, interferon-gamma, tumor necrosis factor, IL-4, IL-6, IL-2, IL-10, IL-12, IL-13, and IL-15 are produced by the lymphocytes with some variation between patients [88]. Thyroid cells express MHC class II and behave as antigen presenting cells (APC). Expressions of ICAM-1, LFA-3, and MHC class I by thyrocytes are enhanced by IL 1, tumor necrotic factor, and interferon-gamma [89]. This response increases the ability of cytotoxic T cell to mediate lysis.

Humoral immunity exacerbates cell-mediated damage both by direct complement fixation (TPO antibodies) and by ADCC [13]. Complement attack initiated via the classic or alternative pathway impairs the metabolic function of thyroid cells and induces them to secrete IL-1, IL-6, reactive oxygen metabolites, and prostaglandins. All of these enhance the autoimmune process [90].

### 24.7. Diagnosis of Autoimmune Thyroid Disease

Diagnosis of AITD is based upon clinical features and supported laboratory investigations. The patient may be euthyroid, hypothyroid, or hyperthyroid depending on the type of disease and the stage of the disease. AITD is detected by measuring circulating antibodies against TPO and TG. A negative test for both antibodies excludes AITD, as 98% of patients are positive for either antibody. TPO Ab is more specific and sensitive than TG Ab in the diagnosis of autoimmune hypothyroidism. Elevated TSH with TPO antibodies is the gold standard for diagnosis of chronic Hashimoto's thyroiditis. TSH Abs that stimulates the TSH-R in Grave's disease is measured to predict neonatal thyrotoxicosis. They can be measured by thyroid receptor assays or bioassays. 

### 24.8. Autoimmune Thyroid Disease and Neoplasms

Thyroiditis and thyroid antibodies are found in a quarter to a third of the patients with thyroid cancer [91]. Pre-existing Hashimoto's thyroiditis is the major risk factor for development of non-Hodgkin's lymphoma of thyroid [92]. Studies have also shown that there is an increased frequency of autoimmune thyroiditis in women with breast cancer [93].

### 24.9. Autoimmune Thyroid Disease and Kidney Disorder

Endocrine abnormalities have been reported in patients with kidney diseases [94]. Thyroid dysfunction causes remarkable changes in glomerular and tubular functions, and in electrolyte and water homeostasis [95]. From a clinical practice viewpoint, it should be mentioned that both hypothyroidism and hyperthyroidism are accompanied by remarkable alterations in the metabolism of water and electrolyte, as well as in cardiovascular function [96].

## 25. Conclusion

Autoimmune thyroid disease occurs as a result of a complex interaction between genetic and environmental factors. The disease occurs due to autoreactive lymphocytes escaping tolerance. Both cell-mediated and humoral responses contribute to tissue injury in autoimmune thyroid disease. Diagnosis of AITD is based upon clinical features and supported laboratory investigations. AITD has been associated with neoplasm and kidney disorders.
